# Effect of Plasma Treatment on the Impact Behavior of Epoxy/Basalt Fiber-Reinforced Composites: A Preliminary Study

**DOI:** 10.3390/polym13081293

**Published:** 2021-04-15

**Authors:** Maria Rosaria Ricciardi, Ilaria Papa, Giuseppe Coppola, Valentina Lopresto, Lucia Sansone, Vincenza Antonucci

**Affiliations:** 1 Institute of Polymers, Composites and Biomaterials, National Research Council, 80055 Portici, Italy; mariarosaria.ricciardi@cnr.it (M.R.R.); vinanton@unina.it (V.A.); 2 Department of Chemical, Materials and Production Engineering, University of Naples Federico II, 80125 Naples, Italy; ilaria.papa@unina.it (I.P.); lopresto@unina.it (V.L.); 3 Institute of Applied Science and Intelligent Systems, National Research Council, 80125 Naples, Italy; giuseppe.coppola@cnr.it

**Keywords:** plasma, basalt, low velocity impact, contact angle, hydrophobic

## Abstract

Hydrophobic surfaces are highly desired for several applications due to their exceptional properties such as self-cleaning, anti-icing, anti-friction and others. Such surfaces can be prepared via numerous methods including plasma technology, a dry technique with low environmental impact. In this paper, the effect of a one-step sulfur hexafluoride (SF_6_) plasma treatment upon the low velocity impact behavior of basalt/epoxy composites has been investigated by using several characterization techniques. A capacitive coupled radiofrequency plasma system was used for the plasma surface treatment of basalt/epoxy composites, and suitable surface treatment conditions were experimentally investigated with respect to gas flow rate, chamber pressure, power intensity, and surface treatment time by measuring the water droplet contact angle of treated specimens. The contact angle measurements showed that treating with SF_6_ plasma would increase the hydrophobicity of basalt/epoxy composites; moreover, the impact results obtained on reinforced epoxy basalt fiber showed damage in a confined area and higher impact resistance for plasma-treated basalt systems.

## 1. Introduction

Natural fibers have been recently studied to replace synthetic fibers for the production of composite materials. Major advantages associated with natural fibers include low cost, low density, high toughness and biodegradability. Among the natural fibers, basalt fibers are attracting significant interest due to good sound insulating properties, good heat resistance (superior to glass), excellent protection from chemical attack, and low water absorption properties [1]. High heat resistance of basalt is useful for applications requiring thermal insulation as for hot fluids transportation pipes. Another fundamental characteristic is the high mechanical performance similar to that of glass fiber that makes this material appropriate to feasibly substitute glass fibers in different mechanical fields like aviation, automotive industry, transportation, and shipbuilding [2,3]. Basalt fiber has higher tensile strength and elongation at failure that can provide good impact resistance and environmental sustainability. Therefore, basalt fibers [4] represent a valid alternative to synthetic ones as reinforcement of composite materials and are currently investigated by several authors. The prominent advantages of these composites include high specific mechano-physicochemical properties, biodegradability, and non-abrasive qualities. However, the hydrophilic nature of natural fibers often results in poor compatibility with hydrophobic polymer matrices, affecting the performance of the resulting fiber reinforced composite. In order to provide good interfacial adhesion and compatibility between the resin and the fibers, the surface can be modified by proper treatments, such as plasma. The use of plasma on polymers is generally studied to develop biosensors [5], microfluidic devices [6,7], food packages [8], systems of drug delivery [9] and enhancing of biocompatibility properties [9,10].

In fact, plasma flow can provide different kinds of physical surface modifications [11] such as ablation, crosslinking, and surface activation. Ablation consists of the removal of surface layers at a molecular level; crosslinking involves the formation of covalent links by the action of two or more radicals, while surface activation induces the increase of the surface energy due to the formation of polar groups.

Recently, the plasma treatment of fiber reinforcement of composites has increased attention for carbon fibers to improve the surface functional groups and physical interaction between the fiber and matrix [12,13]. In fact, Borooj et al. found that plasma treatment increased the roughness of the surface leading to enhanced mechanical interlocking and improved composite interlaminar shear strength (ILSS), due to the inclusion of different polar functional groups such as carboxyl and hydroxyl on the surface of carbon fibers [12]. In addition, a detrimental effect of long exposure time and high power plasma was observed owing partly to damage of the carbon fibers surface and, hence, decrease of the ILSS of treated carbon fiber composites [14].

An important aspect of the plasma treatment is that the material changes are confined to a depth of a few nanometers, reducing the potential of damage to the reinforcement plies. A number of physical processes can occur during plasma treatment such as ablation (cleaning by removing low molecular weight organic contaminants), etching (affecting the surface morphology of the substrate), crosslinking (interconnection of long chain molecules), and surface activation (chemical bonding of reactive molecules with the substrate) [15]. Generally, as already mentioned, the dry fibers are treated by atmospheric plasma before the resin impregnation, in order to increase the hydrophilicity of the fibers that become more compatible with the organic matrix. Thus, plasma treatment improvement enhances the interface adhesion between reinforcement and matrix, which consequently leads to increments of both tensile/flexural strength and dynamic mechanical properties [16,17].

In this study, Sulfur hexafluoride (SF_6_) plasma treatment has been investigated to modify the hydrophobicity characteristics of fiber reinforced basalt/epoxy composites and evaluate the effect of plasma on their low velocity impact behavior. In particular, different parameters of the plasma treatment have been adopted in order to assess the effect on the composite surface and compare the impact properties of both untreated and treated materials.

## 2. Materials and Experimental Set-up

Epoxy basalt fiber composite materials have been realized by using a commercial two-component epoxy resin (Prime 27 by Gurit, slow curing resin with a 100:26 resin/catalyst ratio (density 1.123 g/cm^3^) and a basalt fabric of 200 g/mq (by Basaltex BASALTEX NV, Zuidstraat 18, 8560 Wevelgem, Belgium) by vacuum infusion process technology. After the composite manufacturing and consolidation at 50 °C for 16 h, the composite samples with a (0, 90) stacking and a fiber volume fraction of 0.68, we have obtained the untreated sample called BS neat or BS, from the same basalt fiber composite material we have cut in different pieces (about 15 × 15 cm) that have been treated by SF_6_ plasma in an RIE (Reactive Ion Etching, Angelantoni, Torino, Italy). The SF_6_ gas is introduced into a pressure-regulated plasma chamber at a 100% mass flow ratio using some integrated mass flow controllers which are situated upstream of the plasma chamber and regulate the SF_6_ mass flow to 30 sccm. The mass flow controllers regulate the chamber pressure to 21 Pa (156 mTorr), whereas the roots pump evacuates the chamber downstream at a constant flow rate. After a constant chamber pressure is reached, the plasma is ignited. Afterwards, the prepared samples are stored for one week in an ambient atmosphere.

Three different surface treatment protocols were chosen by varying the time, t, or power, P, of plasma exposure.

Table 1 shows the treatment parameters and the nomenclature for the tested samples.

After the plasma treatment, the unmodified and surface treated composite laminates have been experimentally characterized by performing water contact angle measurements, low-velocity impact tests, and indentation depth measurements on the impacted laminates. In particular, the optical contact angles have been measured by the data physics Optical Contact Angle (OCA) System (The DataPhysics Instruments GmbH headquarters in Filderstadt, Germany).

Impact tests were carried out by a falling weight machine, Ceast Fractovis (Instron 825 University Ave Norwood, MA, US), at different energy levels (U = 10 J, 20 J, 30 J) to perform the so-called indentation tests, useful to study the damage start and evolution. The rectangular specimens, 100 × 150 mm, cut by a diamond saw from the original panels, were supported by the clamping device suggested by the ASTM D7137 Standard and were centrally loaded by an instrumented cylindrical impactor with a hemispherical nose, 19.8 mm in diameter. Tests were conducted using an impactor with a mass equal to 3640 kg that combined with the drop heights allowed us to obtain the selected impact energies.

After the impact tests on the base material and one with the aluminum substrate, the specimens were observed by visual inspection to investigate the damage, whereas a confocal microscope, Leica DCM3D (Leica Microsystems GmbH, Wetzlar, Germany) was used to measure the indentation depth and the roughness of the surface obtained by each plasma treatment. The roughness was analyzed by scanning the tested specimen area for 30 × 40 mm using a Leica confocal microscope. The roughness profile was scrambled for each sample and relative plasma treatment and with a Gauss filter, (a microscope Leica filter) cut-off 0.800 mm. Three measurements were averaged for each condition. The selected roughness parameter is Ra. This parameter indicates the arithmetic mean value of the deviations (taken as an absolute value) of the real profile of the surface with respect to the mean line. This measurement refers to a base length l of the profile analyzed to avoid the influence of other types of irregularities.

## 3. Results

### 3.1. Optical Contact Angle and Roughness Surface Data

**Figure 1:**
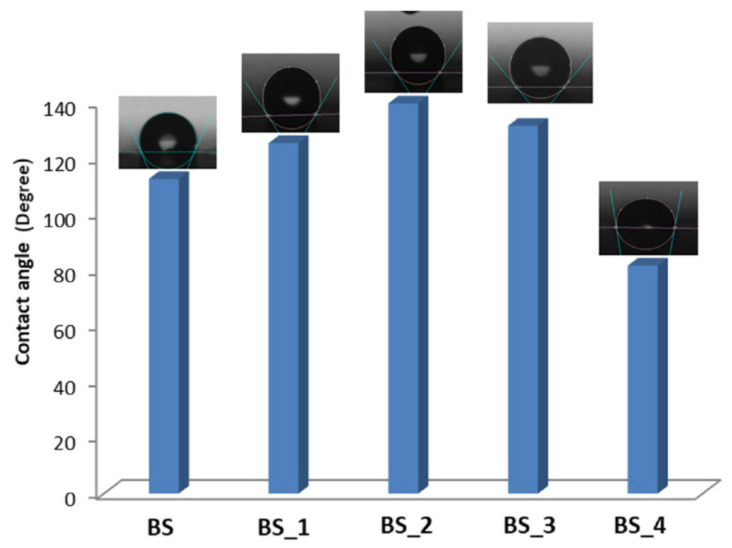
Composite contact angle results.

Each sample has been examined ten times to verify the coating distribution on the surface of the composite. Figure 1 and Table 2 summarize the results and evidence the effect of plasma power and time.

Aside from the observation that higher plasma power results in higher etch rates, it is possible to observe that the sample BS_2 is characterized by a more uniform surface. It has been treated with an average intermediate time between that of composite BS_1 and BS_3 adopting a lower power than that used for BS_4, which is characterized by a substantial variation of the contact angle between 45° and 125°. Thus, a higher value of power doesn’t determine a better distribution of the coating. The outcomes of a plasma treatment depend very strongly on a number of factors such as type of plasma (low- or atmospheric pressure), applied power, and duration of treatment. In the case of sample BS_4, a high power plasma of 300 W has been adopted (see Table 1), finding a not uniform coating or damage of the surface composite. In fact, some parts of the sample showed low contact angle, and others high contact angle. Plasma etching efficiently creates hydrophobic surfaces by two major mechanisms: firstly, by providing sufficient roughness to the surface by the etching process and secondly, by providing a sufficient number of low-energy functional groups [15]. Typically, fluorine-containing plasmas can simultaneously incorporate sufficient amounts of low-energy functional groups as well as creating the desired surface roughness through etching. The resulting main effects of cold plasma treatment are cleaning, ablation or etching, crosslinking, and surface chemical modification, which occur together in a complex synergy, which depends on many plasma parameters.

As reported in Table 2, there is a significant variation of the contact angle as function of the performed surface treatment. In particular, the highest contact angle has been found for the sample BS_2 that was treated with intermediate values of both time and power. However, no roughness variation was observed, indicating an unchanged morphology.

### 3.2. Impact Data

The impact behavior on the neat and treated basalt samples at fixed power and time (155 W, 10 min) were just compared [16] for impact energy, U = 30 J. The results clearly showed that the treatment time affects the material response up to a time t = 20 min. As the surface exposure time increases from 20 to 40 min, no change was recorded by denoting the stability of the surface after treatment with increased time, t.

**Figure 2:**
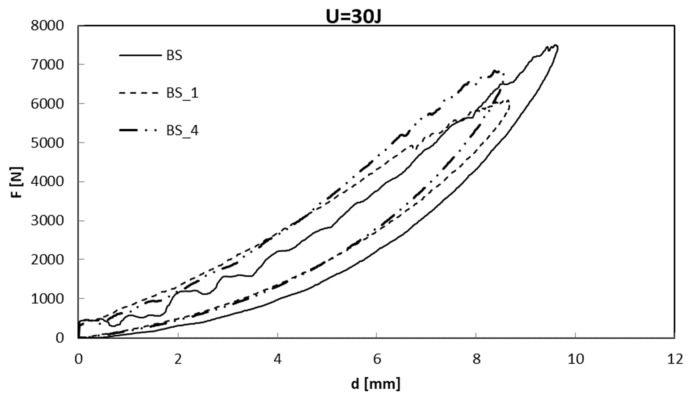
Load-displacement comparison between coated composites, t = 10 min, P = 155, 300 W, (BS_1, BS_4) and neat one (BS).

Thus, it was decided to compare the samples treated at the same plasma exposure time of 10 min and at different power values, BS_1 and BS_4, with neat basalt. These data are shown in Figure 2.

In both cases, the maximum, F_max_, load decreases for the treated composites: among the various used treatment protocols, the lowest maximum load is recorded for BS_1 which was treated by the lowest values of time and power. In the case of the other two composites, BS_2 and BS_3, the maximum load is similarly influenced by the effect of the power and surface processing time variation (Table 3). Fluorine containing plasmas are often used for the surface hydrophobization of materials [18]. During the SF_6_ plasma treatment, fluorine attaches to the composite surface producing OCF_3_, CF_4_, and other fluoride groups, sulfur fluoride (SF_X_) radicals on the composite surface, and the chemical group concentrations depend upon the pressure in the discharge chamber during the plasma treatment. 

The decrease of the Fmax value is determined by the modification of the basalt/epoxy composite chemical structure, because crosslinking surface reactions is promoted by radicals, ions, produced by cold plasma. The crosslinking process not only leads to the mechanical strengthening of the interface, but the crosslinked interface sublayer simultaneously provides an enhanced fiber/polymer interface [19,20,21].

**Figure 3:**
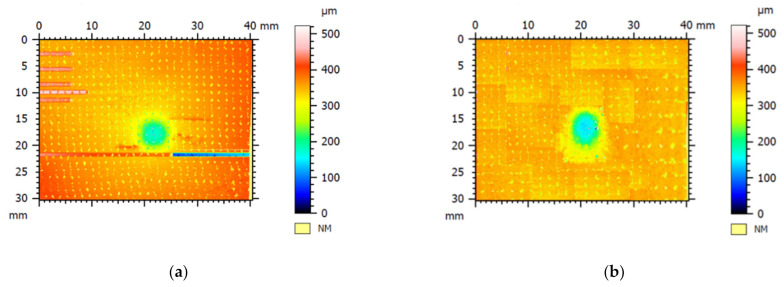
Visual inspection image obtained by a confocal microscope, Leica DCM3D, for the indentation depth measurement of neat (**a**) and coated (BS_2) (**b**) composite sample.

However, thanks to this type of protocol, the BS_2 and BS_4 systems obtain a thicker skin-deep coating which guarantees higher impact resistance [22,23]. It is worth noting that, in the case of the coated materials, the first significant load drop occurs in correspondence to the maximum load, while in the case of neat BS laminates, several load drops, denoting delamination initiation and propagation, are observed. This result means that the treatment influences the damage that for the coated one seems to be localized under the material–impactor contact point in a confined area, as shown in Figure 3.

**Figure 4:**
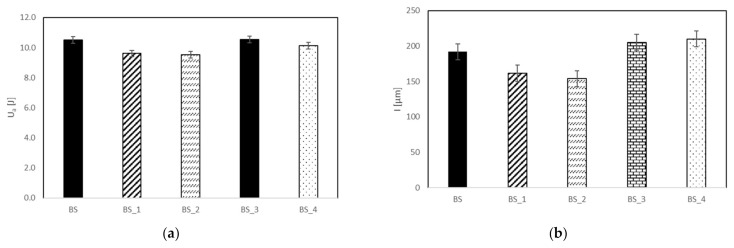
Absorbed energy U_a_ (**a**) and indentation depth, I (**b**), versus different BS systems.

The influence of the coating on the damage mechanism is also confirmed by the lowest absorbed energy, U_a_, and indentation depth, I, measured for the BS treated composite. Reminding ourselves that the indentation depth, I, is the footprint impressed by the impactor on the impacted surface of the specimen (plastic deformation), from Table 3 and Figure 4, it is possible to note that the coated composites BS_1 and BS_2 absorb the same lower impact energy in respect to the other systems, denoting a lower propensity to damage.

This result also corresponds to a lower indentation depth, I. Further, the analysis of the data reported in Table 3 and Figure 4 outlines that the excessive increase in plasma treatment time and power seems to be detrimental for the impact damage.

Thus, the above data induce one to assess that the optimal protocol seems to be that of BS_2, obtained by a fluorine treatment with a power of P = 155 W, and a treatment time of t = 20 min. Thus, for this type of composite, its behavior has been investigated in detail by a complete low-speed impact test campaign at three energy levels, U = 10, 20, 30 J and comparing the results with those of neat basalt composite.

**Figure 5:**
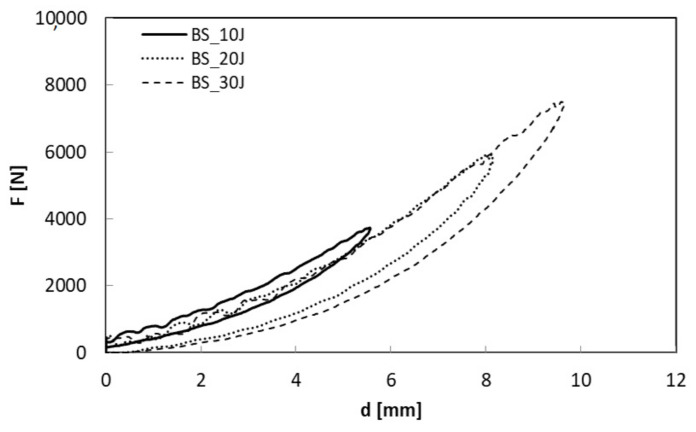
Load displacement curves comparison of neat basalt, BS, at U = 10, 20, 30 J.

**Figure 6:**
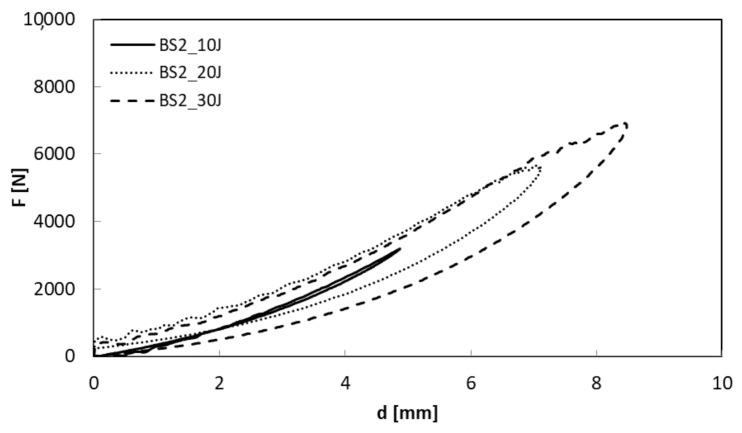
Load displacement curves comparison of treated basalt, BS_2, at U = 10, 20, 30 J.

In Figure 5 and Figure 6, the load-displacement curves at three different energy levels (U = 10, 20, 30 J) for BS_2 and neat basalt, respectively, are shown.

The maximum load increases as the impact energy increases (Figure 5 and Figure 6) even if a small decrease is shown for the treated composite, BS_2 (Table 4).

**Figure 7:**
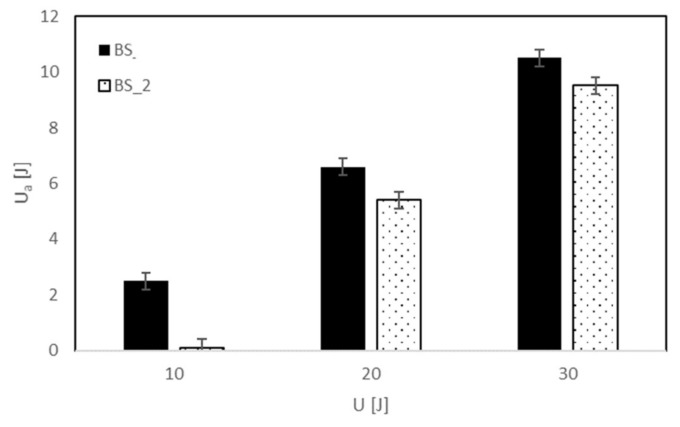
Absorbed energy, U_a_ versus impact energy, U, for neat and treated basalt.

**Figure 8:**
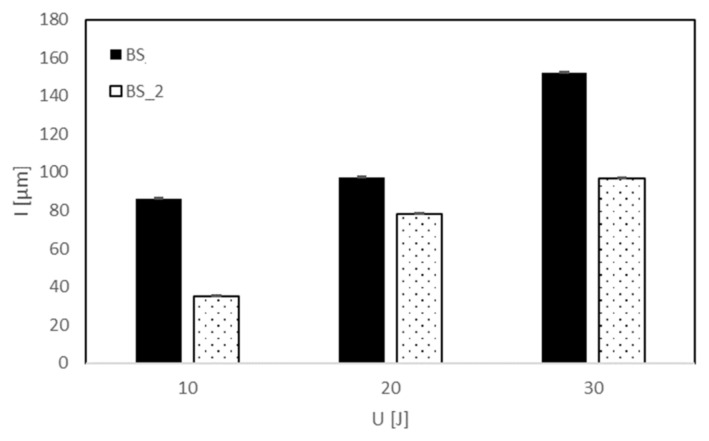
Indentation depth, I versus impact energy, U, for neat and treated basalt.

In particular, in the case of the treated sample impacted at U = 10 J, the curve seems to return on itself, indicating persistent damage. The lowest absorbed energy, U_a_, and the consequent indentation depth, I, confirm the latter result as indicated in Table 4 and reported in Figure 7 and Figure 8.

It is possible to note that the absorbed energy (Figure 7) and the relative indentation depth (Figure 8) for the treated system result are lower than the other for each impact condition. In particular, the indentation seems to reach a threshold value after U = 20 J for BS_2 even if a significative difference of absorbed energy between 20 J and 30 J is detected, indicating that the absorbed energy is dissipated in different modes of damage.

Since the indentation represents the plastic deformation of the laminate surface, it might be thought that the neat composite BS is more prone to this kind of deformation (Figure 8) due to the higher rigidity and maximum load as shown in Figure 2. In conclusion, the modification of the surface chemical structure of basalt/epoxy composite by plasma exposure leads to different mechanisms of energy absorption and deformation.

## 4. Conclusions

The research activity, developed in this work, evaluates the influence of Sulfur hexafluoride (SF6) plasma treatment applied to samples in basalt fiber and epoxy resin. 

The surface hydrophobicity of epoxy basalt composites has been modified by Sulfur hexafluoride (SF_6_) plasma, by investigating the effect of plasma parameters exposure, i.e., time and power.

The measurements of water contact angle evidenced that the increase of plasma time and power can be detrimental for the surface characteristics and the composite impact behavior. Thus, after the selection of the optimal plasma treatment, a complete impact characterization at different energy levels has been performed for the untreated and treated composite subjected to the identified optimal procedure. The effect on the only superficial damage in terms of plastic deformation (Indentation) was evaluated to study the SF6 plasma treatment on the damage entity. The results of the impact tests and indentation measurements on the tested samples showed a significant effect of the surface plasma modification on the impact energy absorption and damage size. In particular, the treated samples evidenced a confined damage, and a lower absorbed energy and indentation depth than those of unmodified composites, confirming the efficacy of plasma treatment to modify the surface properties and the consequent visible impact damage.

## Figures and Tables

**Table 1:** Plasma treatment parameters.

Sample	Surface Treatment Parameter
BS	Neat
BS_1	P = 155 W t = 10 min
BS_2	P = 155 W t = 20 min
BS_3	P = 155 W t = 40 min
BS_4	P = 300 W t = 10 min

**Table 2:** Contact angle and roughness surfaces.

Sample	Contact Angle	Roughness [µm]
BS	112.5° ± 0.13	5.31 ± 0.56
BS_1	125.3° ± 0.08	5.52 ± 0.29
BS_2	139.5° ± 0.10	4.60 ± 0.45
BS_3	131.5° ± 0.15	4.29 ± 0.25
BS_4	81.5° ± 0.62	4.75 ± 0.66

**Table 3:** Impact parameters at U = 30 J.

Sample	Protocol	F_max_ [N]	U_max_ [J]	U_a_ [J]	d [mm]	I [µm]
BS	-	7504.33	30.55	10.50	9.61	181.93
BS_1	155 W, 10 min	6378.02	29.26	9.61	8.58	161.68
BS_2	155 W, 20 min	7165.07	29.36	9.52	8.38	153.93
BS_3	155 W, 40 min	7086.69	29.19	10.54	8.42	205.03
BS_4	300 W, 10 min	7093.22	29.42	10.12	8.54	210.01

**Table 4:** Impact parameters for neat and treated basalt.

	BS		BS_2	
U [J]	F_max_ [N]	U_a_ [J]	F_max_ [N]	U_a_ [J]
10	3737.47	2.49	3794.81	0.10
20	5941.75	6.59	5656.29	5.40
30	7504.33	10.5	7165.07	9.52

## Data Availability

Not applicable.
